# Suggested solutions to barriers in accessing healthcare by persons with disability in Uganda: a qualitative study

**DOI:** 10.1186/s12913-024-11448-4

**Published:** 2024-08-31

**Authors:** Andrew Sentoogo Ssemata, Tracey Smythe, Slivesteri Sande, Abdmagidu Menya, Shaffa Hameed, Peter Waiswa, Femke Bannink Mbazzi, Hannah Kuper

**Affiliations:** 1 Medical Research Council/Uganda Virus Research Institute and London School of Hygiene and Tropical Medicine Uganda Research Unit, Entebbe, Uganda; 2 Department of Global Health and Development, London School of Hygiene and Tropical Medicine, London, UK; 3 International Centre for Evidence in Disability, London School of Hygiene and Tropical Medicine, London, UK; 4 Division of Physiotherapy, Department of Health and Rehabilitation Sciences, Stellenbosch University, Cape Town, South Africa; 5 School of Public Health, College of Health Sciences, Makerere University, Kampala, Uganda

**Keywords:** Solutions, Recommendations, Persons with disabilities, Healthcare access, Uganda, Qualitative

## Abstract

**Background:**

There are 1.3 billion people with disabilities globally, and they frequently face barriers to accessing healthcare, contributing to their worse health and higher mortality. However, little research has explored patient-reported approaches to improve healthcare for persons with disabilities. Consequently, this study aimed to explore possible solutions and recommendations to address the existing barriers to access to healthcare for persons with disabilities in rural Uganda.

**Methods:**

We conducted 27 semi-structured interviews with individuals with various disabilities in rural Luuka district, Eastern Uganda, between September and November 2022. The participants included individuals with visual impairment (*n* = 5), physical impairment (*n* = 5), hearing impairment (*n* = 6), multiple impairments (*n* = 5), intellectual/cognitive impairment (*n* = 5), and albinism (*n* = 1). Interviews were recorded, transcribed verbatim, and thematically analysed. We categorized the solutions using the Missing Billion disability-inclusive health systems framework.

**Results:**

Our findings, framed within the health systems framework, revealed several critical themes. On the demand side, suggested solutions emphasized advocacy and sensitization for persons with disabilities, their communities, and caregivers about healthcare needs. Socio-economic empowerment and community-based health services were also highlighted as essential. On the supply side, participants stressed the importance of training healthcare workers on disability, facilitating dialogue and experience-sharing sessions, and employing health workers with disabilities. Additional recommendations included improving accessibility and reasonable accommodation, organizing inclusive services like special clinic days and outreaches, ensuring representation in health facility management, and establishing comprehensive rehabilitation services with affordable assistive devices.

**Conclusion:**

The multifaceted solutions proposed by persons with disabilities highlight the complex challenges they face in accessing healthcare services and highlight the necessity for comprehensive, sustainable interventions. The call to action for policymakers and healthcare providers is to prioritise the incorporation of disability-inclusive practices and explore multi-dimensional approaches that foster a more inclusive healthcare environment that adequately meets the needs of persons with disabilities.

**Supplementary Information:**

The online version contains supplementary material available at 10.1186/s12913-024-11448-4.

## Background

Universal health coverage (UHC) focuses on ensuring everyone has access to a full range of quality essential health services from health promotion to prevention, treatment, rehabilitation and palliative care without financial hardship [1]. Investing in primary health care (PHC) has been identified as the most effective and cost-efficient way to achieving UHC [2]. However, some people such as people with disabilities are left behind in many aspects of PHC [3]. Therefore, failure to ensure the inclusion of people with disabilities in healthcare will mean that global targets such as UHC will be difficult to achieve [4–6].

People with disabilities experience marginalisation and face lower life expectancy, higher rates of poverty, and reduced access to education and employment opportunities (WHO, 2022), which further limits their access to healthcare services and exacerbates their health conditions [7, 8]. The health and wellbeing of persons with disability is further compounded by the limited resources and infrastructure in low and middle income countries (LMICs), which often lack the necessary healthcare facilities, trained healthcare professionals, and medical equipment to provide adequate healthcare services [9, 10].

People with disabilities experience additional barriers to accessing quality healthcare services, due to inaccessible environments, under-serviced facilities and discriminatory belief systems and attitudes which may hinder their full and effective participation in society [11–14]. Additionally, a recent systematic review on the barriers to accessing primary healthcare services for people with disabilities in low and middle-income countries demonstrated that attitudinal/ belief system barriers, informational barriers, and practical and logistical barriers greatly impact access to primary healthcare services for people with disabilities in LMICs [3]. These barriers deepen inequities in the quality of healthcare provided, and affects the full and equal enjoyment of all human rights and fundamental freedoms of persons with disabilities in line with the Convention on the Rights of Persons with Disabilities [15].

Although barriers to healthcare for persons with disabilities have been studied in other settings, they may not correspond to the lived experiences for persons with disabilities in rural Uganda. Understanding the barriers to accessing health care and how these barriers affect persons with disabilities is particularly important, as such knowledge can inform efforts to address these challenges [3].

There is a lack of solutions to improve access to healthcare for people with disabilities and an evaluation to know what works, to best invest the available meagre resources in order to improve the wellbeing of persons with disabilities [16]. This aligns with the findings of studies in Ghana [17] and the USA [18] underscoring the importance of incorporating recommendations from persons with disabilities in developing effective, evidence-based strategies to effectively address the barriers to healthcare access.

More importantly, identifying possible contextual solutions and recommendations from persons with disabilities is critical for the development of successful interventions aimed at improving health care access and eliminating access disparities, thereby averting further deterioration of health, wellbeing, and functionality [14, 19]. While many studies have primarily focussed on understanding the barriers and facilitators, exploration of the possible solutions and recommendations has been under-researched [17, 18].

However, while there are pockets of good practice and some successful interventions, they are often not widespread and frequently lack full integration of the disability perspective, typically not incorporating the viewpoints of persons with disabilities [20, 21]. Therefore, improving access to healthcare for people with disabilities in these settings requires the examination of possible solutions and recommendations pertinent to development of comprehensive and inclusive healthcare systems that address the unique challenges faced by these populations [17].

### Healthcare system in Uganda

Uganda’s healthcare system is a mixed system comprising public, private, and community-based services, managed primarily by the Ministry of Health [22]. The public sector that provides primary healthcare (first point of contact with the health system) services for the majority of the population is organized into a tiered system, ranging from national referral hospitals to regional, district, and lower level community health centres (HC IV, III, and II), each providing community-based preventive and promotive health services [22, 23]. There are efforts to decentralize services and improve access and healthcare performance through the Uganda’s National Health Policy and Health Sector Development Plan [24], which guide the organization and delivery of health services. However, significant disparities still persist, particularly between urban and rural areas, where healthcare infrastructure is often lacking, and services are underfunded [25]. Vulnerable populations, including persons with disabilities, face additional barriers to accessing care, which are compounded by resource constraints and the uneven distribution of healthcare professionals [3, 12, 26, 27]. Recent health policies and development plans aim to address these issues, but challenges remain in ensuring equitable and effective healthcare delivery across the country [28].

This study sought to explore patient-reported potential solutions and recommendations aimed at enhancing access to and delivery of primary healthcare services for people living with disabilities in rural Luuka district, Eastern Uganda. Uganda is - a low-income country in Eastern Africa with an estimated population of 45.8 million in 2021 with less than 15% of the population living in urban settings [29]. There are wide disparities in health status, underscored by major health system challenges including inaccessible and inequitable service provision to all persons at all times in both the public and private sectors [30].

### Theoretical orientation

**Fig. 1:**
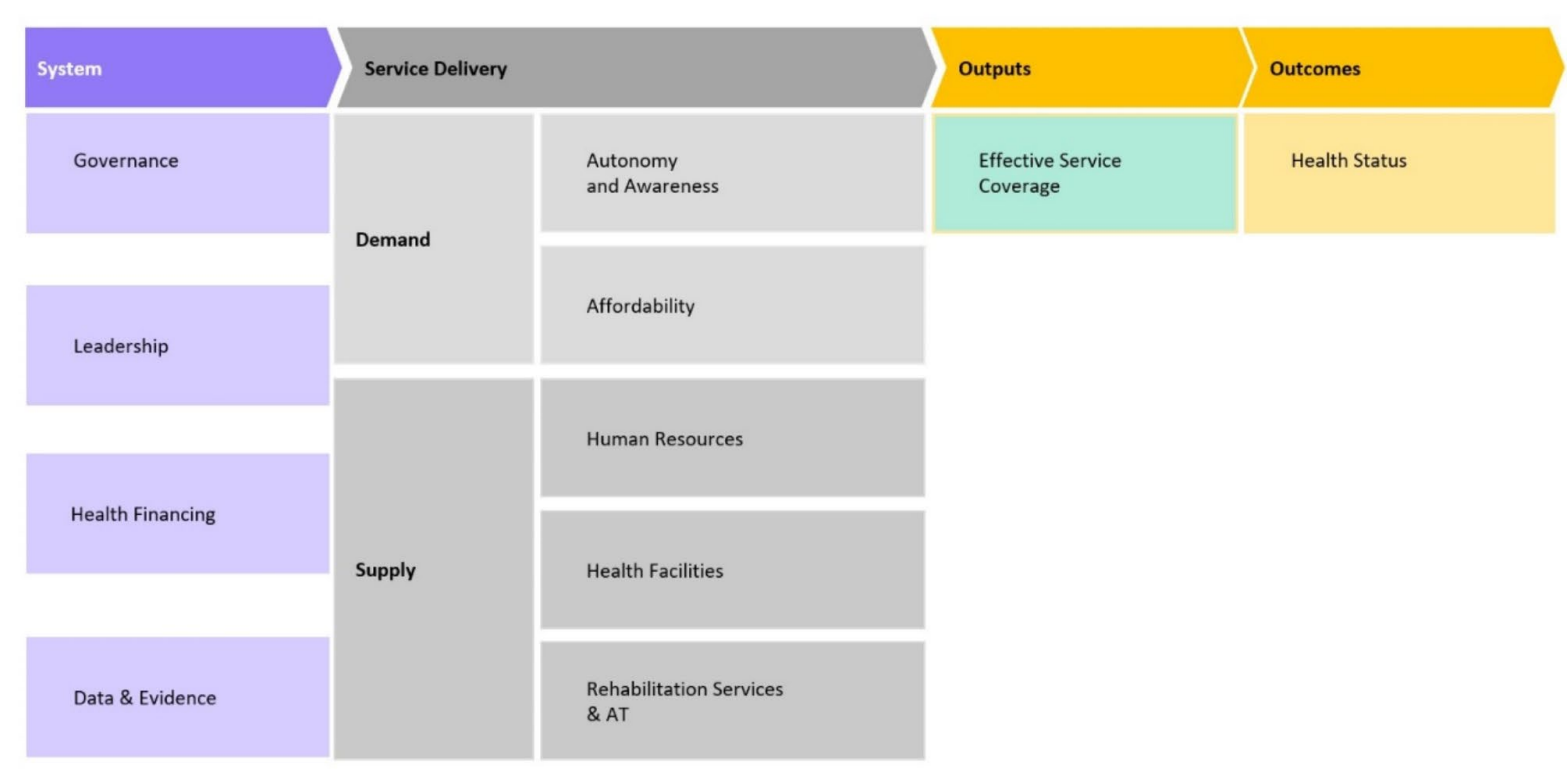
The Missing Billion disability-inclusive health systems framework

To understand the topic, we leveraged our study on the Missing Billion disability-inclusive health system framework [20]. The framework includes 4 system-level components and 5 service delivery components (2 on the demand side, 3 on the supply side) as shown in Fig. 1. The Missing Billion disability-inclusive health system framework delineates key components essential for establishing a robust disability-inclusive healthcare system. We chose this framework because it is relevant for this context as it considers important objectives of disability-inclusive health systems that “expect, accept, and connect” people with disabilities to quality care critical for LMIC settings [20, 31].

## Methods

### Study design

We conducted an exploratory qualitative study as part of the “Missing Billion” project implementation of community-based participatory approaches to improve access to healthcare for persons with disabilities in Uganda [26]. The exploratory qualitative approach [32, 33] was used as our topic of interest is under-researched [17]. Qualitative methods are particularly well-suited to capturing the depth and complexity of individual experiences, providing rich, detailed insights that quantitative approaches might not fully capture [33, 34]. Utilising a qualitative methodology would allow for the exploration of the perspectives and recommendations to improving access to health care for persons with disabilities [32].

### Participants and setting

The study employed purposive sampling to identify people who self-identified as living with disabilities from Luuka district in Eastern Uganda, a region comprising seven sub-counties, one town council, and an approximate population of 203,500 individuals. Participants were purposively selected to represent five distinct impairment categories: physical, hearing, visual, cognitive, and multiple impairments. Recruitment methods included accessing (a) contact lists from the district’s disability focal person, (b) local disability associations, and (c) recommendations from participants involved in the study through a snowballing approach. We identified participants who were able to communicate, demonstrated the ability to give informed consent by correctly answering questions about the study; and lived in the community for more than two years. The sample was diverse in terms of participant age and type of disability. A total of 27 participants aged 18 years or older were approached and agreed to participate (Table 1), with only one individual declining to participate. We determined that our chosen sample size would be sufficient to achieve data saturation (point at which no new data or themes would emerge) [35]. The distribution across impairment categories (Table 1) was as follows: visual impairment (*n* = 5), physical impairment (*n* = 5), hearing impairment (*n* = 6), multiple impairments (*n* = 5), intellectual/cognitive impairment (*n* = 5), and albinism (*n* = 1). We selected a sample size that was large enough and encompassing various impairments to provide diverse perspectives but small enough to allow for an in-depth analysis of each participant’s experiences [35].

**Table 1:** Participants’ profile

Participant No.	Gender	Age	Disability type
P-001	Female	43	Visual Impairment
P-002	Male	50	Visual Impairment
P-003	Male	62	Visual Impairment
P-004	Male	40	Visual Impairment
P-005	Female	60	Visual Impairment
P-006	Female	45	Physical Impairment
P-007	Male	50	Physical Impairment
P-008	Female	20	Physical Impairment
P-009	Male	34	Physical Impairment
P-010	Female	40	Physical Impairment
P-011	Female	30	Hearing Impairment
P-012	Female	44	Hearing Impairment
P-013	Female	21	Hearing Impairment
P-014	Male	21	Hearing Impairment
P-015	Male	37	Hearing Impairment
P-016	Female	26	Hearing Impairment
P-017	Male	30	Multiple Impairments
P-018	Female	18	Multiple Impairments
P-019	Male	23	Multiple Impairments
P-020	Female	80	Multiple Impairments
P-021	Female	25	Multiple Impairments
P-022	Male	18	Intellectual/ Cognitive Impairment
P-023	Male	23	Intellectual/ Cognitive Impairment
P-024	Female	22	Intellectual/ Cognitive Impairment
P-025	Female	20	Intellectual/ Cognitive Impairment
P-026	Male	19	Intellectual/ Cognitive Impairment
P-027	Female	25	Albinism

### Data collection

Between September and November 2022, authors AS and SS, proficient in English, Lusoga, and Luganda, conducted in-depth interviews using a pilot-tested semi-structured interview guide (Supplementary file 1) developed by the researchers in close collaboration with an advisory group of persons with disabilities. The aim was to extract insights regarding potential solutions and recommendations for enhancing access to and provision of healthcare among individuals with disabilities in the region. The researchers (ASS and SS) experienced in disability-related qualitative research, interviewed the participants while participants with hearing impairment were interviewed by a research team member with hearing impairment supported by a sign language interpreter. Each 50–80-minute interview was audio-recorded, with fieldnotes taken by the researchers. The field notes were taken to capture observations, non-verbal cues, contextual details, immediate reflections and any other relevant information that could provide additional context to the verbal data. The interviews with participants with hearing impairment were voiced by the sign language interpreter and recorded. Data collection occurred in private locations, such as participants’ homes, community halls, or health facility compounds, chosen for comfort and confidentiality. Reasonable accommodations were provided to the participants based on their impairment such as large print information sheets, presence of a sign language interpreter, meeting the participant in their homes.

The researchers (AS and SS) held weekly debriefing meetings to compare notes, discuss emerging ideas and generate preliminary findings. This process ensured accuracy in data collection and interpretation, addressing any potential misunderstandings. These meetings continued throughout the analysis phase until data saturation was achieved.

### Data management and analysis

**Fig. 2:**
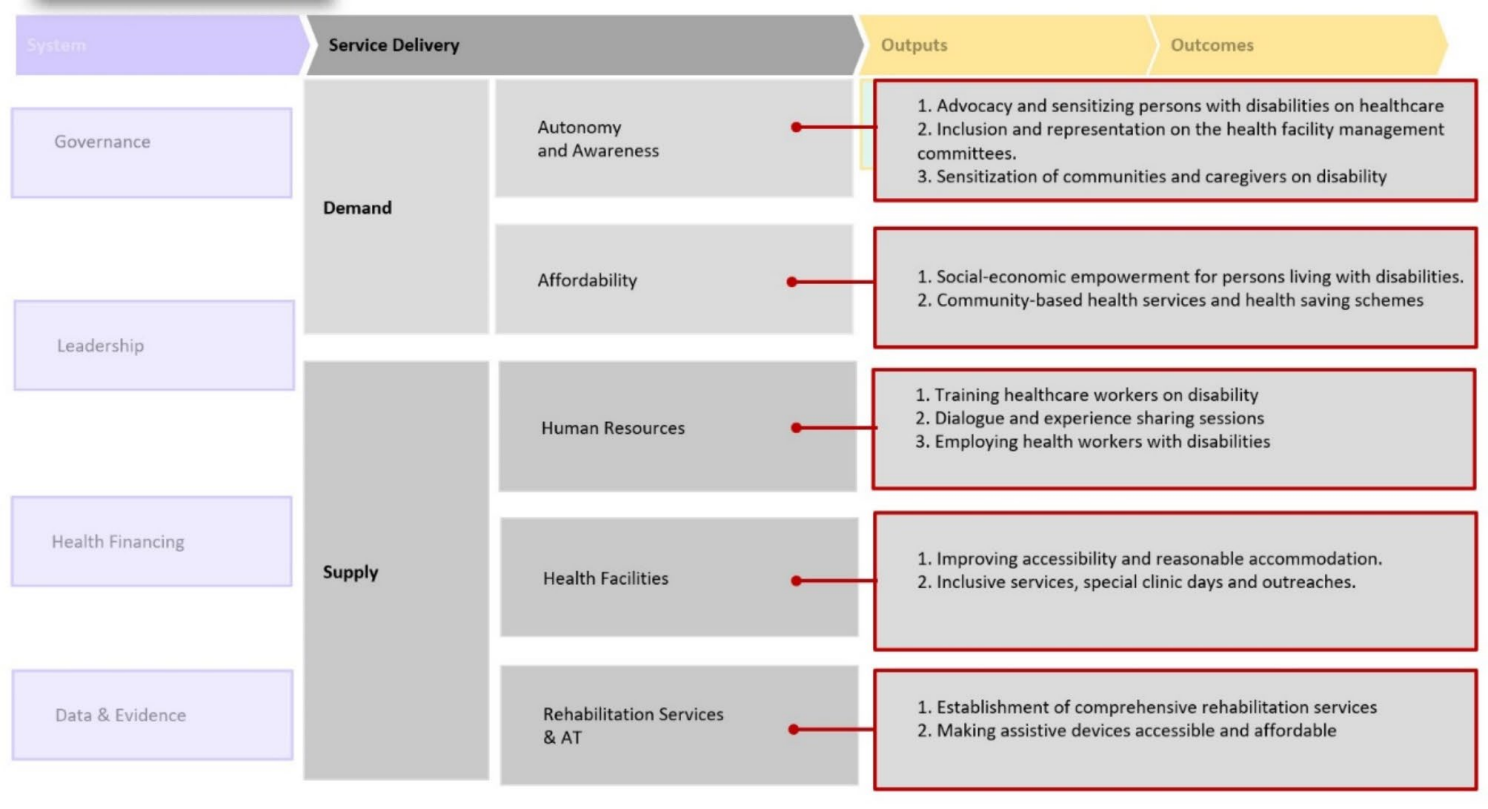
Suggested solutions and recommendations to the barriers to access to health care based on the Missing Billion disability-inclusive health systems framework proposed by the Missing Billion Initiative

All interview recordings were transcribed, with those conducted in Lusoga and Luganda translated into English. Transcripts were then summarized and indexed. Two researchers (ASS and SS) independently manually coded the data using MS Excel. Open coding facilitated the identification of new and evolving themes, while prominent themes raised by participants were identified. After each interview, the field notes were organized and reviewed alongside the transcribed interview data. Key observations and insights from the field notes were integrated into the analysis process to enrich our understanding of the data and to identify themes that may not have been immediately apparent from the interview transcripts alone. By incorporating the field notes into our analysis, we aimed to triangulate the data in order to produce a more nuanced and comprehensive interpretation of the findings, ensuring that our analysis and conclusions accurately reflected the experiences and perspectives of the participants. Thematic data saturation was reached through the analysis of all transcripts, ensuring exhaustion of new codes and themes [35]. Main themes were listed, and illustrative excerpts providing context from participants were reported in the results. Thematic analysis, utilizing a predetermined codebook further refined inductively from emerging themes and based on the Missing Billion disability-inclusive health systems framework [20], was employed to explore responses from persons with disabilities regarding their perspectives on solutions to improving healthcare access. Our focus was on the service delivery components of the framework. These are components that persons with disabilities in the community are very likely to reflect on rather than the system components (see Fig. 2). The framework was instrumental in organizing and categorizing the data during the analysis phase. We used the framework’s components as initial codes or themes, which allowed us to systematically analyse the data, identify patterns and relationships in relation to key theoretical constructs.

### Rigor and trustworthiness

We employed a number of strategies to ensure rigor and trustworthiness in our study. To enhance credibility and validity of our findings, we used triangulation by collecting and analysing data from the interviews and field notes. Additionally, the authors (ASS, SS and AM) had multiple debriefing sessions to discuss emerging themes and ensure a consistent and comprehensive interpretation of the data. At these session meetings, we critically evaluated our biases and assumptions throughout various the study stages.

During purposive sampling of the participants, we considered maximum variation of the sample to include participants with different impairments to ensure relevant and rich data. We maintained the consistency of our coding by ensuring two authors coded the transcripts and any discrepancy, a third author part of the research team supported participated in the coding meetings to ensure dependability. Data collection continued until we reached data saturation, meaning no new themes or insights emerged in the later stages of analysis. We have provided a detailed account of our data collection and analysis methods, as well as a clear description of the study context and participant demographics, to enhance the transferability of our findings.

### Ethics and informed consent details

The research received ethical approval from the Uganda Virus Research Institute’s ethics committee (REC ref GC/127/904) and the London School of Hygiene & Tropical Medicine ethics committee (Ref 26715). Clearance was also obtained from the Ugandan National Council of Science and Technology (Ref SS1348ES) and the Luuka district local government - district health office. Prior to any study related activities, participants were provided information sheet and consent forms in a language they were most comfortable to use (English or Lusoga – local dialect used in the study area). The researchers read and explained the study information to each participant. Participants had the privilege to ask any questions prior to consenting or during data collection. All participants provided written informed consent and for those with cognitive impairment, proxy consent was obtained in addition to guardian consent, in line with ethical guidelines.

## Results

The possible solutions and recommendations related to improving access to healthcare service were categorised based on the service delivery components rather than the system components of the Missing Billion disability-inclusive health systems framework (Fig. 2).

### AUTONOMY AND AWARENESS

*People with disabilities make their own decisions about health care and are aware of their rights and options.*

#### Advocacy and sensitizing persons with disabilities on health care

Participants stressed the need to raise awareness among people with disabilities about their healthcare rights using Village Health Teams (VHTs), healthcare workers, or community meetings. They emphasized the importance of informing them about accessing healthcare services and providing specific guidance on facility visits and service locations.

*“Standing for our rights is key. We need to inform persons with disabilities about their rights. It needs sensitization of people with disability about their health*,* their rights when they get to the health facility. People with Albinism or those with other disabilities should be treated as human beings.”* (Female, 25 years, albinism).

*“We must train and empower persons with disabilities to go to the health facilities when they are sick and not to sit at home and wait to die. They need to know they can also fall sick of other diseases like malaria not related to their disability and they need to go to hospital to receive treatment. I know there are those who think that a lame person like me doesn’t get sick*,* but we have red blood like anyone else*,* so advocacy and training is critical.”* (Male, 50 years, Physical impairment).

Participants emphasized the importance of being empowered to advocate for themselves, boosting their confidence, assertiveness and independence in making informed decisions about their health without reliance on others.

*“We must talk to people in the community*,* we educate and empower them on how to be involved to make informed decisions about their health or services they are supposed to get when they reach the health facility.”* (Male, 40 years, visual impairment).

Additionally, participants indicated the need for peer support and mentorship in the communities and health facilities where individuals with disabilities can connect with others who have similar experiences for advice, emotional support, and encouragement to navigate the healthcare system and advocate for their needs.

#### Sensitization of communities and caregivers on disability

Participants recommended community and caregiver sensitization on disability to raise awareness and understanding of the unique health needs of this population. They suggested that such initiatives could reduce disability-related stigma, discrimination, and negative attitudes, while fostering inclusive and supportive environments that promote the well-being and rights of people with disabilities in accessing healthcare.

*“Yes*,* continue to sensitize the caregivers and communities to support us and not discriminate. We need to be taken to hospital when we are ill.”* (Female, 44 years, hearing impairment).

**AFFORDABILITY -** *People with disabilities can afford to access health.*

#### Social-economic empowerment for persons living with disabilities

Participants highlighted the need to improve their socioeconomic situation through providing opportunities, resources, and support systems to enhance their skills, access economic opportunities, and engage fully in society. They expressed confidence that economic empowerment would enable them to afford healthcare, access assistive devices, and maintain independent living.

*“Our livelihood situation as people with disabilities is not a good. If am empowered to make a living*,* then I can get money to seek care*,* I am able to take care of myself the same way the person who has normal eyes is able to do.*” (Male, 40 years, visual impairment).

Participants noted that empowering persons with disabilities with financial literacy training and resources would not only enhances their financial independence but may also help them to proactively manage their health needs, make informed financial decisions, negotiate payment plans with healthcare providers and meet the costs associated with seeking healthcare.

*“If I could get capital [income to invest]*,* I can carry out maize and coffee business as long as I have capital. This means I can generate money and not depend on others. Therefore*,* I am able to get what I want. Even if it is going to the health facility*,* I will be able to go as I will have the money.”* (Female, 25 years, Multiple impairment).

#### Community-based health services and health saving schemes

Participants suggested that setting up community saving groups for medical and health savings to be able to continuously save small amounts on a regular basis for their health needs and support healthcare access. This would minimise on out-of-pocket cost to which many of them acknowledged not having at the time they need to seek healthcare.

“*We should encourage persons with disabilities to create community savings groups like our group is called “Twisakilala Namukube” and it meets there*,* (pointing across the road). I used to spend my money anyhow then I joined and started saving little by little and because my health is delicate*,* this saving has now helped me meet the hospital bills whenever I could fall sick*,* buying medicines that I needed.”* (Female, 43 years, visual impairment).

Additionally, participants suggested the need for extending health services closer to their communities through community health days and integrated outreaches, encouraging home-based care services to help reduce on costly hospital visits and facilitate timely access to healthcare.

*“I ask the government to bring services close to us. It will make access to health services cheaper. I don’t have to spend on transport or be forced to go to a private hospital. If they are doing community outreaches*,* services would be cheaper and then I don’t need to pay to go to Iganga or Jinja*,* it is very far for me.” (Male 50 years*,* visual impairment)*.

Extending health services to the community was viewed as a mechanism to alleviate the financial burden due to high service and transportation costs due to the long distance to the health facilities, mitigate their inability to meet the costs associated with accessing healthcare, sparing them from resorting to private facilities that pose additional financial strain.

### HUMAN RESOURCES

*Health workforce is knowledgeable about disabilities and has the skills and flexibility to provide quality care.*

#### Training healthcare workers on disability

Participants emphasized the critical need for comprehensive disability training for healthcare professionals. This training aims to augment their understanding, skills, and attitudes towards delivering inclusive and effective healthcare services for people with disabilities, thereby improving healthcare access. By educating healthcare workers on various forms of disabilities, as well as the unique needs, challenges, and rights of individuals with disabilities, this initiative seeks to foster empathy, diminish stigma, and bolster comprehension. Consequently, healthcare workers can offer personalized care that upholds the dignity and autonomy of individuals with disabilities.

*“You need to reach out to the healthcare workers and train them on disability and teach them how to manage us well when we come to access healthcare. Training is key and will greatly help in inclusive healthcare.”* (Female, 40 years, physical impairment).

This disability-inclusive healthcare training is anticipated by interviewees to cultivate an environment conducive to inclusive healthcare delivery.

*“The healthcare workers we have do not know everything. So*,* if there are trainings to help them understand that there is nothing for us without us*,* it will change their attitude*,* the way they see us with disabilities and perhaps become more compassionate when providing care*. (Male, 18 years, Cognitive impairment).

Equipped with enhanced skills and knowledge, healthcare workers will not only exhibit greater confidence in catering to individuals with disabilities but also minimize unnecessary referrals. This, in turn, promises to streamline healthcare delivery processes into culturally competent care, ensuring efficiency and inclusivity across the board.

*“Sensitizing health workers in hospitals about disability to help improve service delivery for people with disabilities. Healthcare workers should be equipped with skills like sign language*,* reasonable accommodation or other key adjustments regarding issues of disabilities.”* (Female, 26 years, hearing impairment).

#### Dialogue and experience sharing sessions

Participants also suggested the establishment of engagements sessions and discussion workshops between health workers and persons with disabilities. The purpose of these sessions would be to listen to and learn in context about the experiences and healthcare needs of persons with disabilities with the aim of addressing stigma, discrimination, stereotypes, and negative attitudes of health workers.

*“First of all*,* most times we are left behind but if they invite us for some workshops and we are able to speak with the healthcare workers on how we feel and how we should be treated*,* the challenges we face*,* maybe it would give an opportunity for the health workers to appreciate what we go through as people who have disabilities.”* (Male, 62 years, visual impairment).

*“Given a chance*,* I am able to engage in discussions and sensitize the healthcare workers very well because I know the challenges a person with disabilities experiences. You should organise that at every health facility there is a seminar led by people with disabilities to teach them [healthcare workers] on how to handle people with disabilities.”* (Female, 18 years, multiple impairment).

Dialogue was seen as a mechanism to support healthcare workers to approach people living with disabilities with sensitivity, and open mindedness, ensuring that their care is delivered with the respect, and consideration.

#### Employing health workers with disabilities

Participants underscored the importance of inclusive recruitment strategies for healthcare workers to foster opportunities for individuals with disabilities within healthcare settings. They stressed the necessity of healthcare workers who not only comprehend the challenges and healthcare requirements of persons with disabilities, but also possess the ability to manage them with empathy and skillful care thereby improving healthcare access.

*“If we have healthcare workers with disabilities*,* they will be an example to other health workers*,* will be advocates for improvements in the health system to make it more accessible and inclusive.”* (Male, 34 years, physical impairment).

Integrating persons with disabilities into the healthcare workforce has the potential to enhance disability awareness among healthcare staff and cultivate a more inclusive and welcoming environment. This, in turn, is expected to encourage individuals with disabilities to seek healthcare services more regularly and confidently.

### HEALTH FACILITY

*Health-care services*,* including health-care facility infrastructure and information*,* are accessible for people with disabilities.*

#### Improving accessibility and reasonable accommodation

Participants strongly advocated for improved accessibility in healthcare facilities, emphasizing the importance of ramps, rails, and accessible pathways for seamless navigation by people with disabilities. They also highlighted the urgent need for accessible restroom facilities, adjustable beds, and information in accessible formats. These measures are crucial for creating an inclusive healthcare environment, enhancing the overall healthcare experiences of individuals with disabilities, and promoting equality in healthcare access.

*“Something needs to be done with our facilities to make them accessible then we can easily come for health service. They should put accessible walkways*,* so we pass with ease. They should have user-friendly toilets. They should bring adjustable beds*,* so it’s easy to get on and off.”* (Female, 40 years, physical impairment).

*“Now most of the hospital beds are very high. We want adjustable beds at the facility so that you can crawl onto the bed independently even in my old age and then it can be adjusted for whatever procedure. This will make life easier for both the patient and the healthcare worker.”* (Male, 80 years, Multiple impairment).

In addition, participants suggested that all health centers needed to be equipped with assistive devices and equipment to facilitate access and enhance the quality of care for persons with disabilities, in order for the healthcare workers to provide adequate reasonable accommodation.

#### Inclusive services, special clinic days and outreaches

Participants suggested the implementation of specific clinic days at health facilities as well as conducting outreaches that prioritize the needs of persons with disabilities. This was with reference to HIV special clinic days. Furthermore, these initiatives would help motivate individuals with disabilities to seek medical care promptly when they are unwell, ensuring dependable and accessible healthcare for this population.

*“What is important is to create priority lines so receive healthcare easily and fast. This also reduces on the time you and the person who has left their work to escort you leave early or make special days for us to get dedicated care. That will make service delivery better.”* (Male, 37 years, Hearing impairment).

Participants proposed creation of necessary accommodations within the broader healthcare system that make the service delivery more accessible and efficient for them rather than isolating care for persons with disabilities.

“*The way we are interested in inclusive service delivery*,* we can’t say that let us have separate health facilities specifically for people living with disabilities*,* that’s not possible apart from us having special clinic days where we are given priority like it is done for HIV or TB or diabetes - they have special days to pick medication from the health facilities. The same can be done for persons with disabilities.”* (Female, 43 years, visual impairment).

In addition, some participants recommended creating dedicated departments with trained staff at health facilities that solely offer services to people with disabilities.

*“Am asking the government to consider us who have disabilities and add more resources at the hospitals for us with disabilities like special departments with trained staff so that I do not have to wait in the long lines. As am crawling in the line*,* it may not be easy for a doctor to see me*,* or I may be run over.”* (Female, 45 years, physical impairment).

Others suggested that the establishment of patient navigation services, scheduling appointments and prioritizing them when they visit health facilities would improve their service experience. Some recommended that health facilities should designate disability focal persons to monitor people with disabilities and make sure they receive timely and appropriate care.

*“They should be able to know that they have people who have disabilities of such a category or those who are in such a condition*,* if that person reaches*,* he will want to be attended to in a special way quickly*,* you never know you may be taking it lightly and it continues to hurt him. But for us*,* a person sees you who is holding the baby and he does not see the child*,* so he continues taking you in a normal way yet when she gets sick*,* she gets into critical conditions.”* (Female, 20 years, cognitive impairment).

#### Inclusion and representation on the health facility management committees

Participants indicated that including people with disabilities on health facility management committees, would promote equal participation in service delivery, decision making processes, contributing to more inclusive and representative healthcare delivery practices.

*“A person with a disability should be included on the health facility committee. That is the only way we can be helped*,* and our voice be heard.”* (Male, 34 years, physical impairment).

Such inclusion was seen as an opportunity to facilitate advocacy for the necessary services required by people with disabilities, promote disability awareness, advocate for policy changes, and improve community-based healthcare services.

### REHABILITATION AND ASSISTIVE TECHNOLOGY

*quality rehabilitation and health services are available.*

#### Establishment of comprehensive rehabilitation services

Participants recommended the setup of rehabilitation services at the health facilities where they can be referred for extensive specialist care including physical therapy, occupational therapy, speech therapy, and psychological support, directly impacting their access to healthcare services. Although participants acknowledged the high costs of these services, the establishment at lower health facilities would minimise on the long distances and costly travel expenses to seek the services elsewhere.

*“we need to promote or invest more in rehabilitation health system*,* so we have some rehabilitation done at least at the health centre IIIs in our district. So*,* besides training healthcare workers there can be a way government can organize routine visits for the health workers to visit rehabilitation centres where they can see what takes place there maybe it will help them gain. (Male*,* 30 years Multiple impairment)*

On the other hand, participants recommended having a streamlined system for connecting persons with disabilities from the primary health care facilities to the more specialist services.

#### Making assistive devices accessible and affordable

People with disabilities strongly emphasized the need for affordable availability of assistive devices such as wheelchairs, hearing aids, glasses, and memory aids. Participants expressed concern that the assistive equipment is prohibitively expensive for individuals with impairments and not readily accessible in the local market. As a result, individuals who require such devices must travel outside of their district to obtain them. The provision of assistive devices at affordable prices would significantly enhance the mobility and independence of people with impairments, thereby improving their overall access to healthcare services.

*“Of course*,* I will talk about transport from home to the facility. Maybe I have this wheelchair*,* but maybe it’s not the type that I would have loved to have. I may need to have the tricycle because that one would be easy for me to get to the facility. Also*,* these assistive devices for the Persons with disabilities are expensive. Just look at this wheelchair*,* it costs UGX 2 million (approx. $530) just a wheelchair yet*,* it my legs*,* it is the one I have to use*,* when you look at the blind*,* the white cane*,* you see it very small but it is UGX 360*,*000 (approx. $96) so things are very expensive. So*,* if the assistive devices are subsidized*,* it would be easy for us to reach the healthcare service points.”* (Female, 40 years, physical impairment).

## Discussion

This study aimed to explore the solutions and recommendations proposed by persons with disabilities in Luuka district, Eastern Uganda, to improve access to healthcare. The analysis identified key solutions related to both reaching (autonomy and awareness, affordability) and receiving care (human resources, health facilities, rehabilitation and assistive technology), encompassing various domains. Firstly, autonomy and awareness are emphasised through advocacy, empowerment, and sensitisation efforts, including informing people about their rights and involving them in decision-making processes. Secondly, affordability is addressed through socio-economic empowerment initiatives, community-based health services, and health saving schemes to minimise financial barriers. Thirdly, enhancing human resources involves training healthcare workers on disability, facilitating dialogue sessions between healthcare workers and persons with disabilities, and advocating for the employment of healthcare workers with disabilities. Fourthly, improvements in health facility infrastructure and services are proposed, including enhancing accessibility and reasonable accommodation measures, implementing inclusive services and special clinic days, and establishing patient navigation services. Finally, addressing rehabilitation and assistive technology needs entails establishing comprehensive rehabilitation services and ensuring the affordability and accessibility of assistive devices. These multifaceted solutions proposed by persons with disabilities aim to address the complex challenges that they face in accessing healthcare services, promoting inclusivity, autonomy, and affordability.

These findings are important to help guide the development and implementation of programmes and policies to improve access to healthcare for people with disabilities. These are important, as a vast body of evidence shows that people with disabilities frequently experience poor health and a range of barriers in accessing services, including evidence from Uganda [3, 7, 8, 36, 37]. Comprehensive analyses from LMICs shows that that the health system is failing to include people with disabilities and the range of interventions to improve the health of people with disabilities in LMICs is targeted at individuals, rather than systemic changes [16].

Our study adds to the understanding of how these potential solutions and recommendations for improving healthcare access can be developed, particularly in low-income settings like Uganda in three substantive ways. We used a qualitative approach and directly engaged persons with disabilities, and so the study provides nuanced insights into their experiences and fundamental changes needed regarding healthcare access [33, 34]. This firsthand perspective is invaluable for addressing context-specific barriers and tailoring interventions accordingly. Moreover, the study’s focus on community-based participatory approaches underscores the importance of grassroots involvement in designing and implementing solutions [38, 39]. This is particularly relevant in resource-constrained settings where top-down approaches may be less effective, but also aligns with the disability movement slogan of “Nothing about us, Without us” [39]. A twin-track approach actively involving persons with disabilities that focusses on mainstreaming disability, tailored and targeting people with disabilities is required in implementation of interventions and solutions that address the barriers to healthcare access for persons with disabilities [27]. The study also highlights the importance of addressing not only physical accessibility but also socio-economic factors such as empowerment, financial literacy, and social support networks in enhancing healthcare access for people with disabilities. This holistic approach acknowledges the multifaceted nature of barriers to healthcare and emphasizes the need for comprehensive, sustainable interventions.

We note that participants suggested creation of special clinic days that may improve access, provide dedicated time and resources tailored to their specific needs, offer specialized services and reasonable accommodations to better meet the needs of persons with disabilities. However, we acknowledge that this approach may have negative consequences such as unintentionally reinforcing segregation rather than integrating them into mainstream healthcare services, stigmatization or marginalization if they are only able to access healthcare services on designated special clinic days, rather than being able to access services every day. The special clinic days may also require designating additional resources and staffing, which could divert resources away from efforts to make healthcare services universally accessible and inclusive on all days.

### Strengths and limitations of study

The strengths of our study include the first qualitative exploration of solutions and recommendations to healthcare access improvement in rural Uganda. We interviewed participants with various impairments representative of the disabilities people experience. However, their suggestions may not always be practical or applicable - such as training all healthcare workers on sign language. Therefore, their views must be balanced by other pieces of evidence and triangulated with data from other stakeholders. The qualitative methodology enabled a detailed exploration of perspectives of persons with disabilities. Some of the data were collected by person with disability which aided effective data collection. The utilisation of the service delivery components of the Missing Billion disability-inclusive health systems framework [20] to guide study that includes consideration from both the demand and supply side provided a comprehensive understanding of the topic. The framework supports a structured approach to assessing the holistic inclusion of persons with disabilities into the health system, leveraging on key indicators related to different components. The limitations of this study include the rural setting, which may not reflect and differ from perspectives of participants from an urban cosmopolitan setting. The participants suggested possible solutions and recommendations that may need other factors in combination to cause improvement in healthcare access for persons with disabilities. Participants were representative of one district. However, as they were with various impairments and recruited using several strategies, they may inform overall perspectives of persons with disabilities in a rural setting. As participants were purposively selected, there is a possible influence of social desirability bias on participant responses.

### Implications: research, service provision

The findings from our study suggest the need for future studies to explore possible solutions and recommendations in other regions of Uganda or similar low-income settings. Additionally, there is a need for research to develop, test and implement interventions in addressing the barriers to healthcare access. There is not one “magic bullet” to overcome the barriers to healthcare faced by people with disabilities, and so we recommend that studies should explore multi-dimensional approaches. Our study suggests valuable insights for policy makers and program implementers, emphasising the significance of integrating disability-inclusive practices into healthcare service delivery, such as adapting health facilities and training healthcare personnel, to promote health equity for individuals with disabilities. Additionally, we advocate for resource allocation and support for legislation that protects the rights of persons with disabilities towards improving accessibility and affordability of healthcare services.

## Conclusion

This study underscores the imperative of integrating disability-inclusive practices into healthcare service delivery to ensure health equity for persons with disabilities. The multifaceted solutions proposed by persons with disabilities highlight the complex challenges they face in accessing healthcare services and emphasise the need for comprehensive, sustainable interventions. Moving forward, there is a call to action for policymakers and healthcare providers to prioritise the incorporation of disability-inclusive practices, allocate resources, support legislation protecting the rights of persons with disabilities, and explore multi-dimensional approaches to overcome barriers to healthcare access.

### Electronic supplementary material

Below is the link to the electronic supplementary material.

Supplementary Material 1

## Data Availability

The data analysed during the current study are provided within the manuscript. The transcripts and codebook are not publicly available due to the potential for identifying individual participants.
