# Supplementary material for: Suggested solutions to barriers in accessing healthcare by persons with disability in Uganda: a qualitative study
Source: BMC Health Serv Res. 2024 Aug 31;24:1010. doi: 10.1186/s12913-024-11448-4 (PMC11366160; doi:10.1186/s12913-024-11448-4)
Supplement: Supplementary file 1 — Supplementary Material 1 [file 12913_2024_11448_MOESM1_ESM.pdf]

Qualitative Interview Guide for people with disabilities

The Missing Billion: Using participatory approaches to improve access to healthcare for disabled people in Uganda.

Objective: To find out about people's experience accessing healthcare, common issues, and advice on establishment of participatory groups

These questions should be used to guide discussion but do not have to be used in the sequence listed below. The interviewer should follow up on any additional issues that may arise and seem important in relation to the issues above.

Introduction

- Greet them and thank them for their time
- Identify yourself by name and organisation.
- Read out the information sheet. Remind them of confidentiality and anonymity. Check if they have any questions. Remind them that they are free to decline to answer any of the questions or stop the interview at any time.
- Record their consent/assent in the relevant form OR record verbal consent.
- Start recording

Table 1. To be completed for each participant	
Participant Code	
Interview date and time	
Interview location or mode (phone, video)	
Interviewer	
Interviewee<br><br>*(Record caregiver name if they are present)*	
Nature of impairment(s)	
Gender	
Age	
General observations (anything which might impact how the interview is conducted)	

Background

1. Could you tell me about your disability (or health difficulties) please?
   Prompts:
   - How would you describe your disability? For example, what are the difficulties you experience in day to day life?
   - Do you use any assistive devices to help you?
     a. If no, would you benefit from these, or do you have access to these?
   - Who helps you with daily activities if needed?
   - Please describe your household situation. Who do you live with?

Experience accessing general healthcare

2. Can you tell me about the last time that you were unwell?
   Prompts:
   - Can you describe to me how you felt? What was the matter?
   - What did you do try to make it a bit better?
   - [If the person describes seeking healthcare, move on to question 3]
   - [If the person did not describe seeking healthcare]:
     - Could you tell me more about why you decided not to go to a clinic or hospital?
     - [If the person didn't seek care]: Can you tell me about the last time that you went to a clinic or hospital?

3. [If person sought care]: Can you tell me about this time that you went to a clinic or hospital?
   Prompts
   - What made you decide to go?
   - How did you get there? What did that involve?
   - where do you normally receive care?
   - Interaction in facility: Did you get the care that you wanted or needed?
   - What was the best and worst aspects of the experience?

4. Have you received any services from a Village Health Worker or Community Health Worker?
   Prompts
   - What service did you receive?

Now I would like to ask you some questions about your experiences in receiving health services. For these, please think back to the last few times you've received these services

5. In general, are you able to get healthcare when you need it? Can you explain your answer?

6. What do you think could be done to improve your experience of health care?
   Prompts:
   - Awareness

- Accessibility
- Affordability
- Attitudes

Community group experiences and preferences

7. What other kinds of help would be useful for you to take good care of your health
   Prompt
   - what would help you in maintaining your health?
   - what would help you in seeking health when ill?

8. Have you ever been involved in a community group of people with disabilities? For instance, self-help groups or parent groups? If so, can you describe this to me?
   Prompt
   - What motivated you to attend the group?
   - How often did you meet?
   - What did you like or not like about the group?
   - Are you still in the group? If so, why? If not, why not?

9. We are planning to start community groups of people with disabilities. This group would meet regularly to share experiences (good and bad)- we hope this could be a way to try and improve health and access to health care for people with disabilities.
   - Would you be interested in joining a group like that?
   - What would encourage you to join?
   - What would discourage you from joining?

   If this group was set up in your village or a nearby village, what would be the difficulties, and how could we overcome, including:
   - People with different types of impairments
   - People with different ages
   - Same sex or mixed sex groups
   - Open to carers or anyone else with a genuine interest

10. What factors should we consider when organising the groups?
    Prompts
    - Frequency of meeting: How often should they meet? How often would be convenient?
    - Length of meeting: What length of time should the group meet for?
    - Place of meeting: Where should they meet?
    - Leadership: Who should lead it?
    - Are there any other factors?

11. Thank you for taking the time to speak to us today. Is there anything else you would like to add before we finish? Any comments or questions?
